# Cognitive Development in Children of Mothers with Hypertensive Disorders During Pregnancy: Findings from the ALSPAC Study

**DOI:** 10.1192/j.eurpsy.2025.354

**Published:** 2025-08-26

**Authors:** B. Dachew, G. Ayano, R. Alati

**Affiliations:** 1 School of Population Health, Curtin University, Perth, Australia

## Abstract

**Introduction:**

While the immediate physical health consequences of hypertensive disorders during pregnancy are well-documented, their potential impact on children’s mental health and cognitive outcomes remains relatively under-investigated. Research exploring the link between hypertensive disorders during pregnancy and cognitive function has yielded conflicting findings. Some studies report associations, while others fail to establish a link or even suggest a protective effect. Many of these studies have focused on children with intrauterine growth restriction, low birth weight, small for gestational age, and/or preterm births—factors already known to influence cognitive development. Furthermore, almost all of this research focuses on early childhood, leaving a critical gap in our understanding of the long-term effects into adolescence, a period characterised by rapid cognitive development and academic achievement.

**Objectives:**

This study aimed to examine the associations between hypertensive disorders during pregnancy and intelligence quotient (IQ) in children at the ages of 8 and 16 years.

**Methods:**

Our study sample comprised participants in the Avon Longitudinal Study of Parents and Children (ALSPAC) cohort, an ongoing population-based longitudinal birth cohort in Bristol, Avon, United Kingdom. Children’s IQ was measured using the Wechsler Intelligence Scale for Children (WISC-III). This study included over 4900 and 3300 mother-child pairs at ages 8 and 16, respectively. Binary and multinomial logistic regression analyses were used to estimate odds ratios for the associations.

**Results:**

Hypertensive disorders of pregnancy (gestational hypertension and/or pre-eclampsia) were not found to be associated with lower IQ scores in children at ages 8 and 16. In the multinomial logistic model, we found children born to mothers with gestational hypertension but not pre-eclampsia were more likely to have above-average IQ at age 16 compared to average IQ children born to mothers without gestational hypertension (OR = 1.42; 95% CI: 1.03 – 1.94). This association did not persist when children with below-average IQ were used as the reference category in the analysis, and no such associations were also observed at the age of 8 years.

**Conclusions:**

Our findings revealed no evidence of associations between hypertensive disorders during pregnancy and lower IQ scores in children ages 8 and 16. The observed association between gestational hypertension and higher odds of having an above-average IQ at age 16 needs further investigation. Our findings were based on a cohort study with a longer follow-up period, offering a higher level of evidence than previous studies. The consistency of our findings across different developmental stages strengthens the validity of our results and suggests that any potential effects of hypertensive disorders during pregnancy on cognitive function may be limited or transient.

**Disclosure of Interest:**

None Declared

